# Changes in sensorimotor network activation after botulinum toxin type A injections in patients with cervical dystonia: a functional MRI study

**DOI:** 10.1007/s00221-018-5322-3

**Published:** 2018-07-03

**Authors:** Martin Nevrlý, Petr Hluštík, Pavel Hok, Pavel Otruba, Zbyněk Tüdös, Petr Kaňovský

**Affiliations:** 1 Department of Neurology, University Hospital and Faculty of Medicine and Dentistry of Palacký University, I. P. Pavlova 6, 775 20 Olomouc, Czech Republic; 2 Department of Radiology, University Hospital and Faculty of Medicine and Dentistry of Palacký University, Olomouc, Czech Republic

**Keywords:** Functional MRI, Cervical dystonia, Botulinum toxin, Brain plasticity

## Abstract

Botulinum toxin type A (BoNT) is considered an effective therapeutic option in cervical dystonia (CD). The pathophysiology of CD and other focal dystonias has not yet been fully explained. Results from neurophysiological and imaging studies suggest a significant involvement of the basal ganglia and thalamus, and functional abnormalities in premotor and primary sensorimotor cortical areas are considered a crucial factor in the development of focal dystonias. Twelve BoNT-naïve patients with CD were examined with functional MRI during a skilled hand motor task; the examination was repeated 4 weeks after the first BoNT injection to the dystonic neck muscles. Twelve age- and gender-matched healthy controls were examined using the same functional MRI paradigm without BoNT injection. In BoNT-naïve patients with CD, BoNT treatment was associated with a significant increase of activation in finger movement-induced fMRI activation of several brain areas, especially in the bilateral primary and secondary somatosensory cortex, bilateral superior and inferior parietal lobule, bilateral SMA and premotor cortex, predominantly contralateral primary motor cortex, bilateral anterior cingulate cortex, ipsilateral thalamus, insula, putamen, and in the central part of cerebellum, close to the vermis. The results of the study support observations that the BoNT effect may have a correlate in the central nervous system level, and this effect may not be limited to cortical and subcortical representations of the treated muscles. The results show that abnormalities in sensorimotor activation extend beyond circuits controlling the affected body parts in CD even the first BoNT injection is associated with changes in sensorimotor activation. The differences in activation between patients with CD after treatment and healthy controls at baseline were no longer present.

## Background

Cervical dystonia (CD) is the most common form of focal dystonia, characterized by involuntary sustained contractions of neck muscles resulting in an abnormal rotation or tilt of the head in specific directions (Stacy 2000). The pathophysiology of CD and other focal dystonias has not yet been fully elucidated. Results from neurophysiological and imaging studies suggest a significant contribution of the basal ganglia and thalamus in the development of focal dystonias (Peterson et al. 2010). Recently, it has become clear that the role of the basal ganglia extends beyond motor control into cognitive and sensory functions as well as into sensorimotor integration (Tinazzi et al. 2009a, b). In the last few years, an increasing number of studies have also presented the cerebellum as another important subcortical brain structure in patients with dystonia (Filip et al. 2013a, b, 2017). Finally, other functional imaging and electrophysiological experiments suggest functional abnormalities in the premotor and primary sensorimotor cortical areas together with aberrant sensorimotor integration, which is considered to be a crucial factor in the development of focal dystonia (Tinazzi et al. 2009a, b; Hinkley et al. 2009; Opavský et al. 2011, 2012). However, the published studies differ in terms of observed hypo- and hyperactivation in these cortical areas. Differences among task conditions, including testing of dystonia-affected and unaffected body parts, can partly explain this variance.

Further important insights into the pathophysiology of focal dystonias have come from studies investigating the effects of botulinum toxin (BoNT) treatment. BoNT is currently considered to be one of the most effective therapeutic options in the management of focal dystonias (Jankovic 2004). Undoubtedly, the introduction of the first-generation BoNT products not only led to a breakthrough in dystonia treatment but also to advances in dystonia research. We now know that the dystonic hyperactive and cholinergically sensitive extrafusal fibers as well as the intrafusal muscle fibers are the prime targets of BoNT therapy (Rosales and Dressler 2010). It is the effect of BoNT in muscle spindles that would eventually modify proprioceptive spindle afferents, as these are partly dependent on the intrafusal muscle fiber tensions. A modification of the central programs with BoNT may eventually occur at the spinal and supraspinal levels (Rosales and Dressler 2010). The clinical effect of BoNT on dystonia is, therefore, assumed to be mediated by dynamic changes at multiple levels of the sensorimotor system, from the neuromuscular junction up to the cerebral cortex, as documented by the previous behavioral and electrophysiological studies (Kaňovský et al. 1998; Abbruzzese and Berardelli 2006). The previous functional magnetic resonance imaging (fMRI) studies from our center showed significant treatment-related changes in the sensorimotor network in patients receiving long-term treatment with BoNT type A (Opavský et al. 2011, 2012).

Nevertheless, specialists in movement disorders clinics soon realized that dystonia may behave differently over the course of BoNT treatment. The first reports described the changes of the muscular pattern (Gelb et al. 1991; Deuschl et al. 1992; Marin et al. 1992, 1995; Kaňovský et al. 1997) that may have also implied a central mechanism of dystonia.

We assume that the changing clinical behavior and evolving clinical response to BoNT treatment will also be reflected in changes of task-related functional MRI activation after therapy. The aim of the presented work is to study changes in the sensorimotor network in patients after the very first BoNT injection, using the same task as in our previous work (Opavský et al. 2011).

## Subject and methods

Patients enrolled in the project underwent a comprehensive neurological examination by a movement disorders specialist. All subjects had typical clinical symptoms for at least 12 months and underwent polyelectromyographic examination of neck muscles. To be eligible for the study each patient had to have magnetic resonance (MR) imaging of the brain with no structure abnormality. Each patient was informed in detail about the goal and the course of investigation, and signed an informed consent form. The study protocol was approved by the local ethics committee, in accordance with the principles and recommendations of the Declaration of Helsinki, 1975 and later revisions.

Twelve BoNT-naïve patients (1 male and 11 females; aged 48.8 ± 11.7 years, range 31–70 years) with CD were examined with fMRI during a skilled hand movement with their eyes closed. The examination was repeated 4 weeks after the first BoNT injection to the dystonic neck muscles. Twelve age- and gender-matched healthy controls (2 males and 10 females; aged 49.7 ± 13.9 years, range 25–64 years) were examined using the same functional MRI paradigm without BoNT injection.

The severity of CD was evaluated using the Toronto Western Spasmodic Torticollis Rating Scale (TWSTRS) (Consky and Lang 1994) at two sessions: at week 0, on the day of screening, of the first fMRI examination before the BoNT injection, and at week 4, on the day of the second fMRI examination. In all patients, the injected muscles were determined on the basis of a polyelectromyographic examination, provided by 4-channel Keypoint workstation, Medtronic®, Minneapolis, MN, USA. The details of the electromyographic examination and BoNT injection were described in our previous work (Kaňovský et al. 1998). All patients were treated with onabotulinum toxin type A (Botox^®^; Allergan, Inc, Irvine, CA, USA) in concentrations of 25 IU/ml. The demographic and clinical data of the patients are presented in Table 1.

**Table 1:** Demographic data of the patients (both CD and control group) and results of TWSTRS before and after BoNT-A injection

Control group		Study group				
Sex	Age (years)	Sex	Age (years)	Total BoNT-A dose (Botox U)	TWSTRS at week 0	TWSTRS at week 4
F	52	F	45	200	19	10
M	59	F	45	200	15	8
F	34	M	60	150	10	4
F	25	F	42	150	24	19
F	26	F	56	150	18	9
F	55	F	40	200	17	7
F	64	F	64	100	16	6
F	61	F	33	200	19	12
F	62	F	55	100	13	7
M	57	F	44	200	15	8
F	46	F	70	100	13	7
F	55	F	31	200	12	7
Mean	49.7	Mean	48.8	162.5	15.9	8.7

Prior to the imaging session, participants were trained in the laboratory in the active task to be performed in the scanner. The task was a complex sequential opposition of individual fingers to thumb with the following order of movements: index finger 1×, ring finger 2×, middle finger 3×, and little finger 4×. During fMRI scanning, patients had their eyes closed, and instructions to start and stop movement were given verbally in MR-compatible headphones. In a block paradigm, movement (7.5 s) was alternated with rest (7.5 s). Each experimental run consisted of 16 movement-rest block pairs, for a total of 4 min. Experimental conditions were repeated twice with the same hand. Performance was visually monitored, recording the number of finger sequences completed per block.

MR imaging data were acquired on 1.5 T scanners (Avanto and Symphony, Siemens, Erlangen, Germany) with a standard head coil. The MR imaging protocol covered the whole brain and included anatomical T_1_-weighted images to provide an immediate overlay with functional data, fluid-attenuated inversion recovery (FLAIR) images to visualize brain lesions, functional T_2_*-weighted (BOLD) images during task performance and rest, and a high-resolution 3D anatomical scan (MPRAGE). BOLD images were acquired using gradient-echo echo-planar imaging (EPI) sequence, with repetition/echo time (TR/TE) = 2500/40 ms, field of view (FOV) 220 mm, and 30 axial slices, to provide 3.4 × 3.4 × 5 mm resolution. A total of 96 images were acquired per each 4-min functional run. The subject’s head was immobilized with cushions to assure maximum comfort and minimize head motion.

Prior to fMRI analysis, the imaging data of patients with the left-sided dystonia leading muscle were flipped in the left–right direction (Johansen-Berg et al. 2002). FMRI data processing was carried out using the FSL version 5.0 (FMRIB’s Software Library, http://www.fmrib.ox.ac.uk/fsl) (Smith et al. 2004; Jenkinson et al. 2012). The following pre-statistics processing was applied: motion correction; slice-timing correction using Fourier-space time-series phase-shifting; non-brain removal; spatial smoothing using a Gaussian kernel of full-width at half-maximum (FWHM) 8 mm; grand-mean intensity normalization; high-pass temporal filtering with sigma 7.5 s; and spatial normalization/registration to the standard-space MNI template. Time-series statistical analysis was carried out using a generalized linear model, implemented in FMRIB’s improved linear model (FILM) with local autocorrelation correction. Group analysis was performed using FMRIB’s Local Analysis of Mixed Effects (FLAME) stages 1 and stage 2 with automatic outlier detection. Statistical maps were thresholded using clusters corrected *P* = 0.05. The voxelwise *Z* (Gaussianized T) threshold was adjusted to reflect the expected effect size. We evaluated (1) mean activation thresholded at *Z* > 3.5; (2) within-group differences in patients at *Z* > 2; and (3) between-group differences at each timepoint and within-group changes over time at *Z* > 1.7. The differences were evaluated within the respective significant clusters of mean activation.

## Results

All patients were injected into the muscles identified by polyelectromyography. The dose for each cervical muscle was 50 IU, and the mean total dose for a patient was 162.5 ± 43.3 IU. The significant clinical effect of BoNT injections was evaluated using the TWSTRS at week 4. The mean value of TWSTRS at week 0 was 15.9 ± 3.8, and at week 4, it was 8.7 ± 3.8 (*P* = 0.00002, one-sided paired *t* test). Details are provided in Table 1. All patients and controls were right-handed, and conventional brain MRI was completely normal in all subjects. For the hand motor task, patients used the hand ipsilateral to the dystonic leading muscle. In 7/12 patients, it was the dominant hand, and in the remaining 5/12 cases, the non-dominant one. Subjects in the control group used hand randomly. The proportion of dominant vs. non-dominant hands was the same in both groups.

**Fig. 1:**
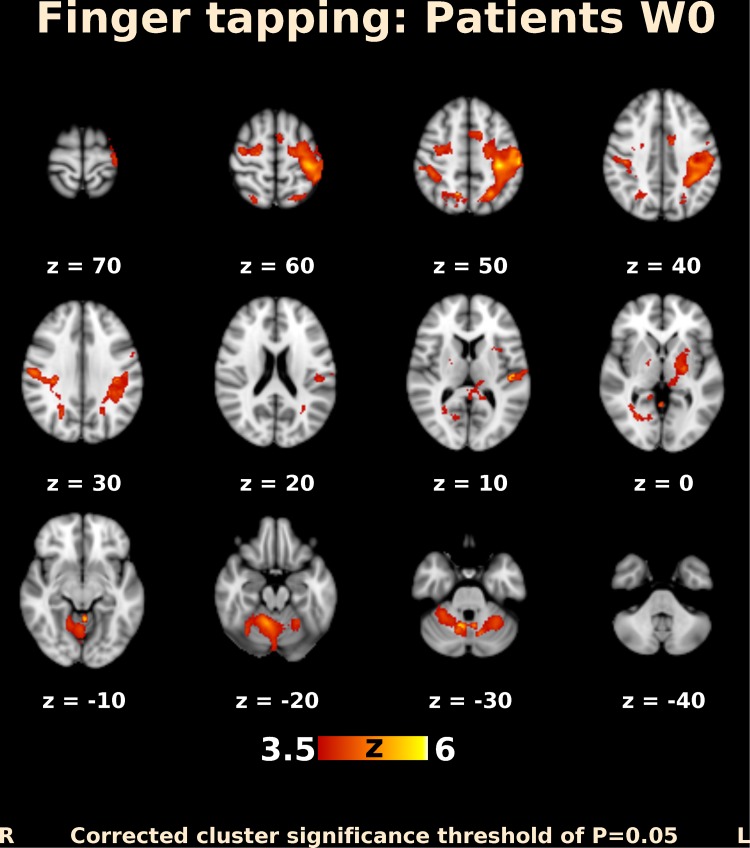
Functional MRI activation map in patients with CD before BoNT-A injection. Slices are labeled with *Z*/*Y* coordinate in standard MNI152 space

**Fig. 2:**
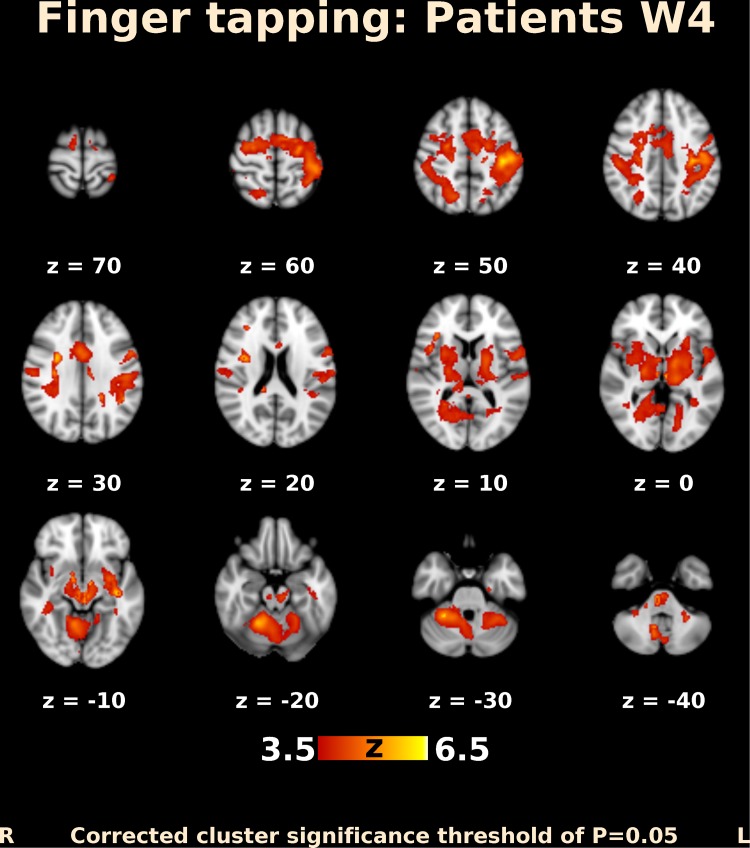
Functional MRI activation map in patients with CD 4 weeks after BoNT-A injection. Slices are labeled with *Z*/*Y* coordinate in standard MNI152 space

**Fig. 3:**
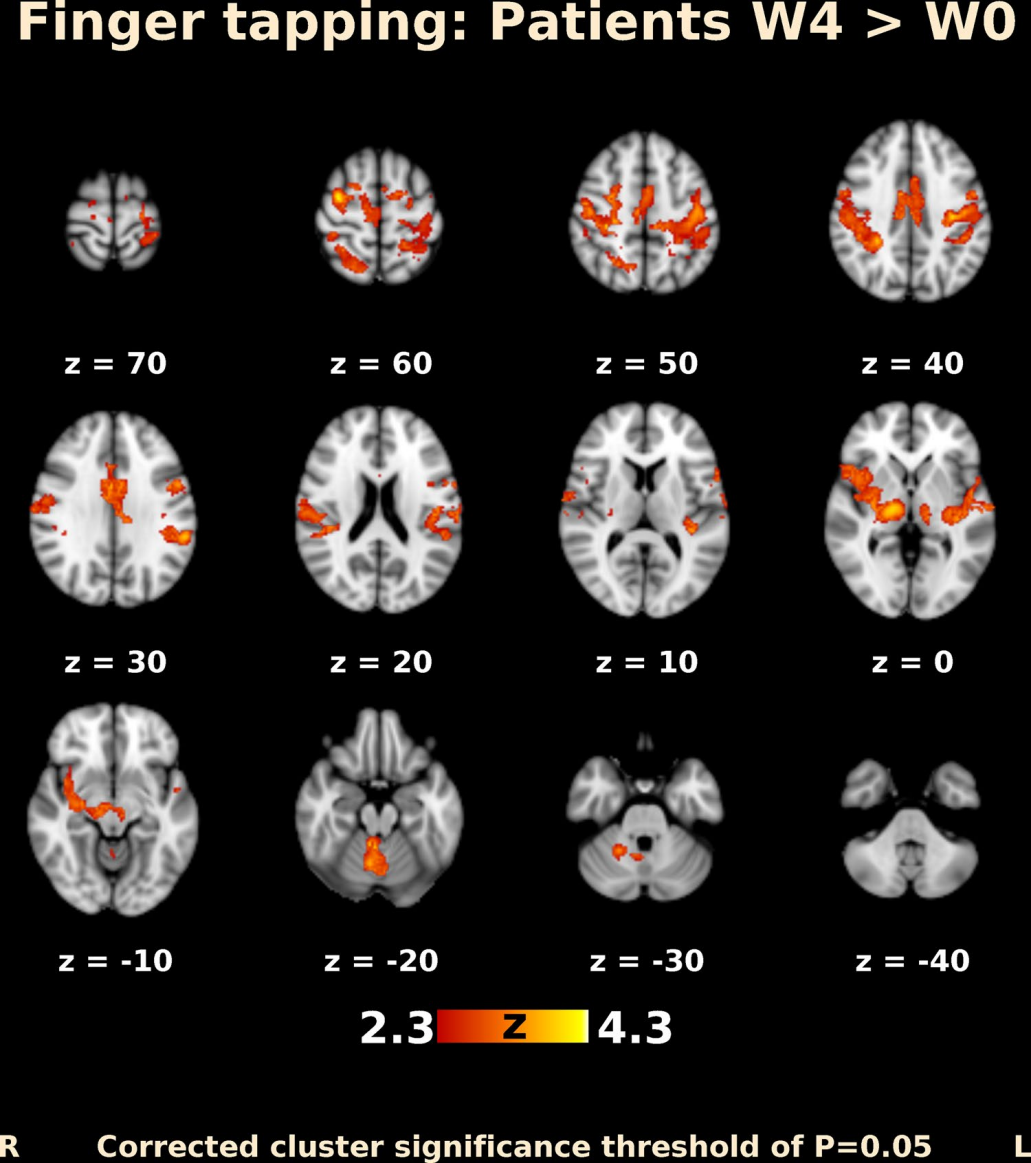
Functional MRI activation map (transversal slices) in patients with CD. Differences in activation after and before BoNT-A injection. Slices are labeled with *Z*/*Y* coordinate in standard MNI152 space

**Fig. 4:**
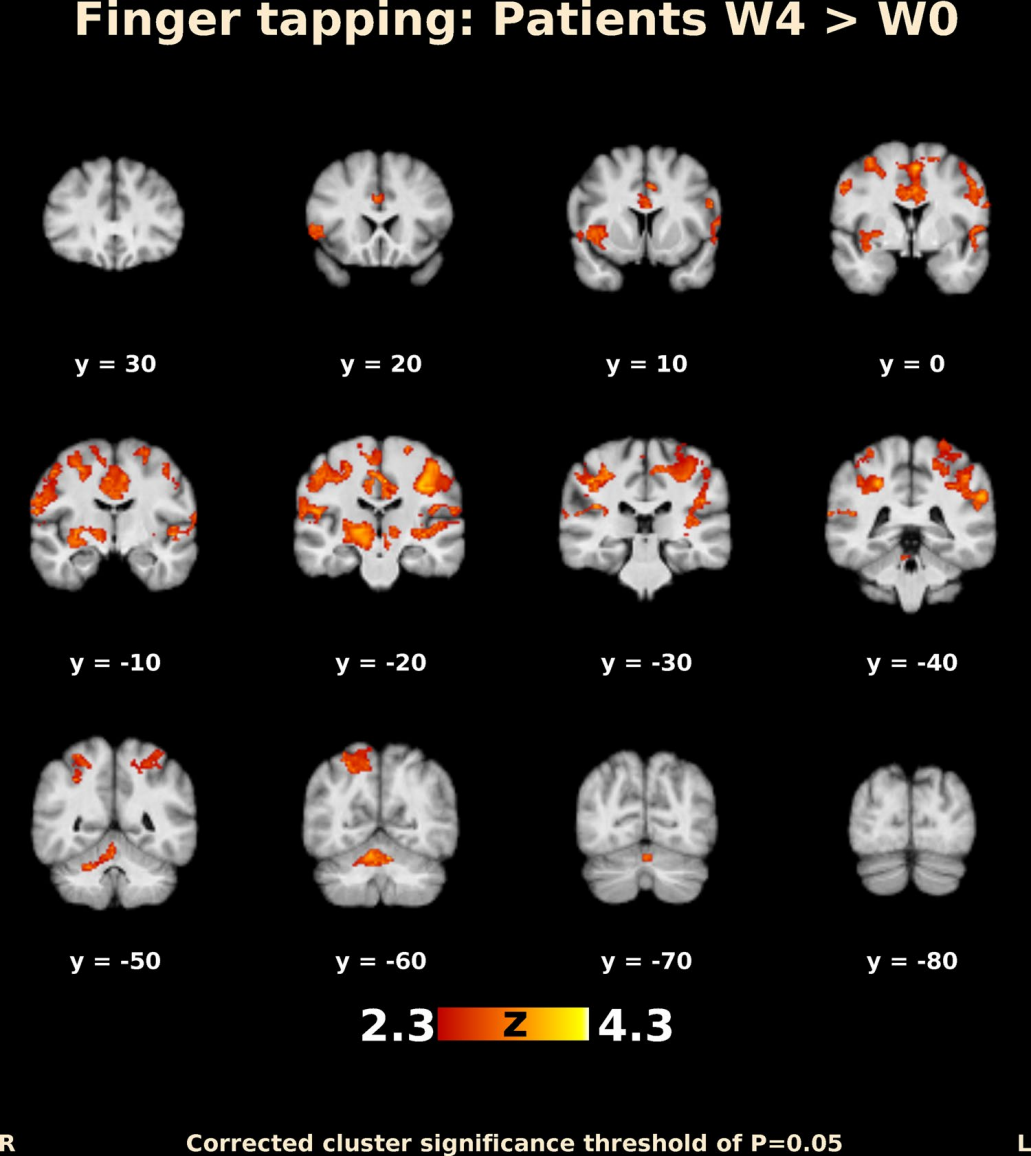
Functional MRI activation map (coronar slices) in patients with CD. Differences in activation after and before BoNT-A injection. Slices are labeled with *Z*/*Y* coordinate in standard MNI152 space

**Fig. 5:**
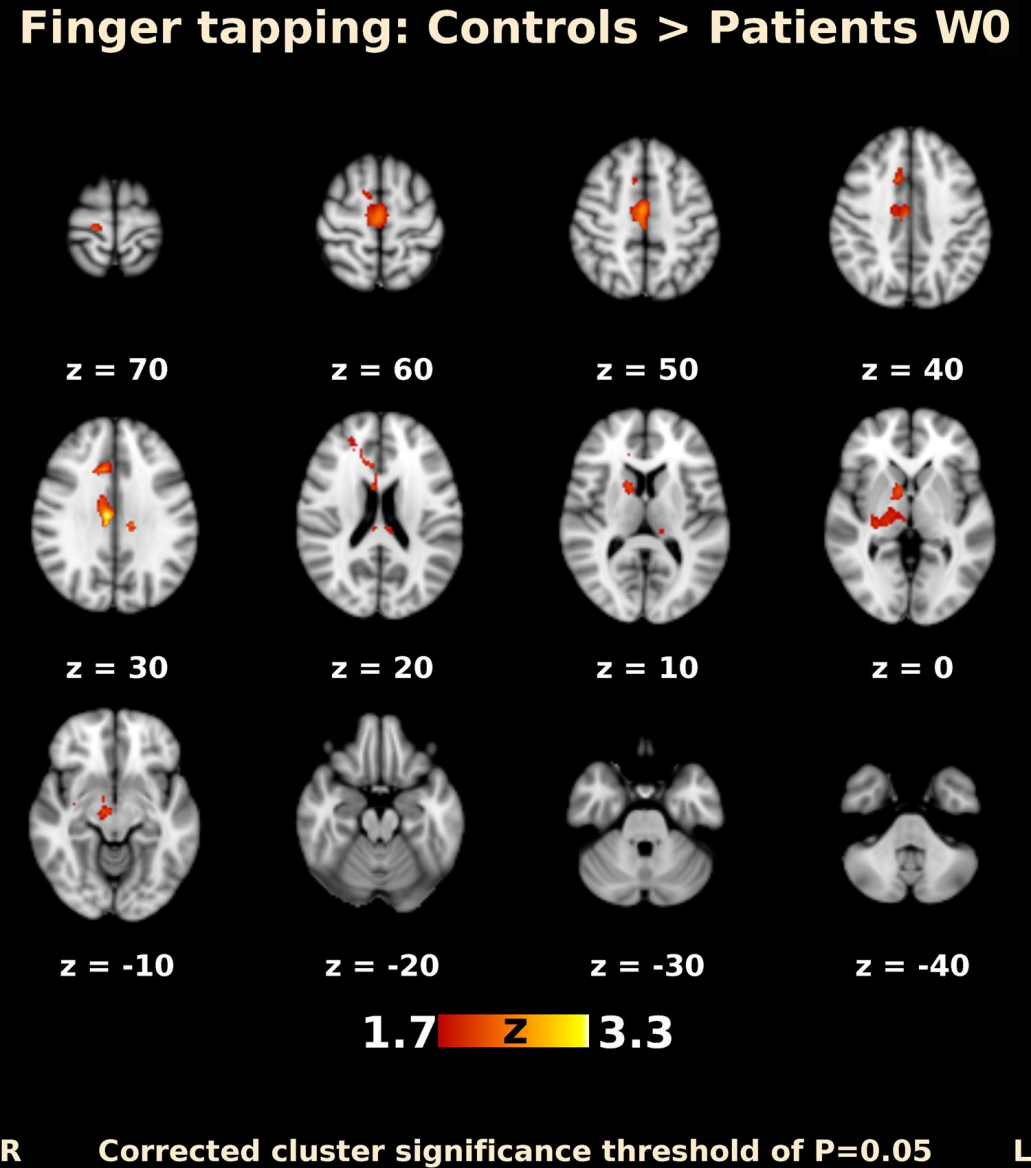
Functional MRI activation map (transversal slices). Differences in activation between CD patients group before BoNT-A injection and control group. Slices are labeled with *Z*/*Y* coordinate in standard MNI152 space

Before BoNT injection, patients performing finger movements activated multiple brain areas, predominantly within the sensorimotor system, including the contralateral primary motor and somatosensory cortex, contralateral secondary somatosensory cortex, bilateral premotor cortex, contralateral supplementary motor area (SMA), bilateral superior and inferior parietal lobule, bilateral cerebellum, contralateral thalamus, bilateral pallidum, and putamen (Fig. 1). The activation map at week 4 after BoNT injection showed a more extended but similar distributed brain network (Fig. 2). A direct comparison of both timepoints (paired contrast) revealed that the activation increased after treatment in most of the brain areas activated before BoNT injection, especially in the bilateral primary and secondary somatosensory cortex, bilateral superior and inferior parietal lobule, bilateral SMA and premotor cortex, predominantly contralateral primary motor cortex, bilateral anterior cingulate cortex, as well as in the predominantly ipsilateral thalamus, insula, and putamen. A significant increase in activation was also apparent in the central part of cerebellum, close to the vermis (Figs. 3, 4); however, there was no significant activation decrease after BoNT injection. When compared to the control group, patients before treatment showed significantly lower activation mainly in the bilateral SMA, ipsilateral cingulate and paracingulate cortex, as well as in the ipsilateral caudate, pallidum, and thalamus (Fig. 5), whereas there was no significant difference between the control group and the patient group after BoNT injection. No significant movement artifacts (maximal framewise movement head displacement was 2.28 mm in one subject; in the rest, it was smaller than 2 mm) were found in any of the MRI images.

The possible influence of faster movements was correlated with the expected task-related hemodynamic response function, which could potentially negatively affect presented results. Evaluating the amount and influencing of task-correlated motion were extracted six original motion parameters (three rotations and three translations) and two derived motion parameters (the absolute voxel displacement from a reference volume and the relative voxel displacement between two consecutive volumes), which were estimated in each subject during the preprocessing. Next, Spearman correlation coefficient was used to correlate each motion vector with the task vector convolved with the hemodynamic response function. The absolute values of the correlation coefficients were compared between the sessions using pairwise sign rank Wilcoxon test. As a result, none of the tested coefficients differed significantly between the sessions (*p* = 0.2 or greater). The overall correlation coefficients were rather low (ranging from *ρ* = 0.08 to *ρ* = 0.19). Thus, we consider any potential effect of task-correlated motion to be negligible.

## Discussion

In this work, we have studied changes in fMRI activation after the first BoNT injection. We consider this trait of the study population as one of the significant contributions of our study, since most of the previous papers reported either changes in long-term-treated patients with CD (Carbon et al. 2008; Obermann et al. 2008, 2010; Opavský et al. 2011, 2012; Burciu et al. 2017) or focused on differences between treated patients and controls, rather than on effects of therapy (de Vries et al. 2008). In BoNT-naïve patients with CD, BoNT treatment was associated with a significant increase of activation in finger movement-induced fMRI activation of several brain areas, especially in the bilateral primary and secondary somatosensory cortex, bilateral superior and inferior parietal lobule, bilateral SMA and premotor cortex, predominantly contralateral primary motor cortex, bilateral anterior cingulate cortex, ipsilateral thalamus, insula, and putamen, and the central part of cerebellum, close to the vermis. These results support the previous observations that the BoNT effect has a correlate at the central nervous system level (e.g., Kaňovský et al. 1998; Šenkárová et al. 2010; Palomar and Mir 2012). The abnormal cortical activation detected during skilled motor tasks performed with a non-dystonic body part also confirms the previous electrophysiological and functional imaging observations that sensorimotor abnormalities in the dystonic brain extend beyond the directly clinically affected sensorimotor representations (Kaňovský et al. 2003; Thickbroom et al. 2003; Opavský et al. 2011, 2012).

In cervical dystonia, earlier fMRI studies by Opavský et al. (2011, 2012) showed significant changes in the sensorimotor network in patients receiving long-term treatment with BoNT. The results of the present study in patients after the first BoNT injection show certain similarities, especially with respect to the localization of the activation changes. However, in contrast to the decrease of activation in long-term-treated patients reported by Opavský et al. (2011, 2012), the present results demonstrate an increase of activation in patients with CD after the very first BoNT injection. Although the limitation of a small patient cohort has to be acknowledged, the opposite direction of activation changes occurring in almost the same brain areas in a single type of focal dystonia in response to either initial or long-term BoNT therapy could provide evidence for long-term brain plasticity and more complex changes induced by BoNT, which involve not only the neuromuscular junction, but also the central nervous system.

The significant treatment-induced changes in our study were detected in areas involved in sensorimotor control and motor learning. In the following section, we will review the function of these regions and discuss their role in the pathophysiology of CD and in the response to BoNT treatment.

The SMA, which was hypoactivated in CD and showed activation increase after BoNT treatment, is considered to be involved in many processes such as posture regulation, internal generation of movement, bimanual coordination, and movement sequencing (Tanji 1996; Chouinard and Paus 2010). In primates, dystonia models demonstrated SMA hyperexcitability, an abnormal increase of proprioceptive inputs to the SMA, and wider sensory receptive fields and a mismatch between sensory inputs and motor outputs (Cuny et al. 2008). These observations may suggest that abnormal sensory inputs coming to SMA neurons participate in the development of dystonia. Hyperexcitability may then decrease the demand for recruitment of SMA neurons to control voluntary movement, which would manifest as reduced task-related activation in functional MRI. BoNT treatment supposedly reduces the abnormal afferent input, thereby reducing the baseline hyperexcitability of the SMA. After treatment, voluntary movement may, therefore, require increased engagement of SMA neurons. Such adaptive increase in activation of the medial premotor cortex has been demonstrated in response to many pathological processes, such as stroke, injury, etc. (e.g., Kantak et al. 2012).

The cingulate cortex is another structure that showed significant hypoactivation in patients with CD and treatment-related activation increase. It is a structurally heterogeneous brain region involved in emotional, cognitive, and motor tasks (Torta and Cauda 2011). The dorsal cingulate sulcus has several motor regions that are active during movement. The cingulate cortex has rich anatomical connections with SMA and both structures are implicated in integration of emotional and motor processing (Oliveri et al. 2003). Therefore, the presented changes in the cingulate cortex probably reflect a similar mechanism as the changes in SMA.

Further intriguing treatment-related activation changes were detected in the central and pericentral parts of the cerebellum. Although the cerebellum is not traditionally noted among the major substrates for development of dystonia, interest in this neuronal structure has increased recently (Avanzino and Abbruzzese 2017,2012; Sadnicka et al. 2012; Filip et al. 2013a, b) as its role in the pathophysiology of dystonia has been suggested by animal models (Jinnah et al. 2005; Raike et al. 2005; Vidailhet et al. 2009), imaging studies (Carbon et al. 2008; Obermann et al. 2010; Opavský et al. 2011, 2012; Prudente et al. 2016; Burciu et al. 2017), neurophysiological studies (Liepert et al. 2004), and even analyses of secondary CD (LeDoux and Brand 2003; Extremera et al. 2008). Neurological disorders originating from the cerebellum (e.g., ataxia) are usually associated with a loss of function. However, different syndromes can arise from the same pathway as different defects alter the output in different ways. In dystonia, it is still disputable whether the cerebellum is the source of the disease or just a node in a complex network trying to compensate for dysfunction of other parts of the brain.

The activation increases post-treatment and was also observed in the secondary somatosensory cortex, which is located in the parietal operculum. It is implicated in higher order functions in somatosensory processing, but it is also believed to integrate information from the two sides of the body, and to participate in visuospatial attention, learning, and memory. According to the previous electrophysiological and imaging evidence, CD seems to be associated with disorders of not only motor but also sensory cortical processing, perhaps at the level of sensorimotor integration (Siggelkow et al. 2002; Abbruzzese et al. 2001; Frasson et al. 2001; Rosales and Dressler 2010). Abbruzzese and Berardelli (2003) consider the aberrant sensorimotor processing to be a key factor for the development of focal dystonias. In a broader sense, the sensorimotor integration involves all parts of the motor and sensory system, including the motor circuits, in which the basal ganglia and the premotor and motor cortex are the principal components.

Finally, the hypoactivations and treatment-related activation increases were also detected in the ipsilateral striatum, pallidum, and thalamus. The involvement of subcortical structures is not unexpected as some previous studies in CD-reported abnormal bilateral activation of the basal ganglia and thalamus during non-dystonia-associated tasks (Obermann et al. 2008; Opavský et al. 2011). Moreover, the internal pallidum serves as a target for effective modulation of CD and other forms of primary dystonias using deep brain stimulation, with an imprecisely characterized mode of action. Whereas higher field MR scanners have certainly provided better spatial and temporal resolution, our spatial resolution of 3.5 × 3.5 × 5 mm appears sufficient to reliably detect basal ganglia activation in normal subjects and neurological patients (Obermann et al. 2008; Hok et al. 2017; Marchal-Crespo et al. 2017).

With respect to overall pattern and direction of activation changes, the previous studies in long-term-treated patients with CD reported that CD was associated with hyperactivations before the BoNT injection (Obermann et al. 2008; Opavský et al. 2011), whereas our study in untreated patients demonstrates widespread hypoactivations. Similar activation decrease was documented previously in a heterogeneous group of eight patients with CD, where five of them were BoNT-naïve (de Vries et al. 2008) and also in another focal dystonia, writer´s cramp (Castrop et al. 2012). However, the global picture seems to be even more complex, since a recent study utilizing fMRI in CD during a force production task reported both distributed activation increases and decreases in comparison with healthy controls (Burciu et al. 2017). Although the provided evidence is difficult to reconcile, the differing direction of functional changes (increased vs. decreased activity compared to healthy controls) may be explained by differences in patient populations, especially the differences in treatment [e.g., naïve vs. long-term-treated patients (Obermann et al. 2008; Opavský et al. 2011)], and functional MRI activation tasks [sequential finger opposition (Opavský et al. 2011) vs. forearm contraction (Obermann et al. 2008) vs. wrist flexion/extension and fist clenching (de Vries et al. 2008)].

Both in neurophysiology and functional imaging, cortical differences between baseline HC and patients with dystonia diminish following a successful treatment with BoNT. The implication is that a peripheral blockade of effectors may influence the central motor programs in dystonia. As we await more data on the probable ‘direct’ retrograde effects of BoNT (e.g., Antonucci et al. 2008), the ‘indirect’ effects remain tenable to date, the latter being hinged upon the normalization of abnormal muscle-spindle functioning in dystonia (Rosales and Dressler 2010). The consequent and apparent normalization of the cortical disorder following BoNT injections in dystonia as observed in neurophysiological studies may indicate that the manipulation of proprioceptive afferent input has a substantial impact on the disorder directly at the central level (Kaňovský et al. 1998; Gilio et al. 2000; Kaňovský and Rosales 2011). It is important to emphasize that treatment with BoNT leads to changes in the central nervous system not only in dystonia, but also in spasticity, as shown in the previous fMRI (Šenkárová et al. 2010; Veverka et al. 2012; Tomášová et al. 2013) and transcranial magnetic stimulation (Huynh et al. 2013) studies. We are aware that there are similarities as well as differences in the two BoNT indications. Dystonia reflects maladaptive plasticity, whereas patients with stroke manifest both adaptive changes related to recovery of function and maladaptive changes likely underlying spasticity. BoNT aims to specifically targets the maladaptive process in both conditions. The results of these studies showed a much more complex effect of the long term and regular BoNT injections, although the pathophysiology of spasticity differs from pathophysiological processes in dystonia (Veverka et al. 2016).

We acknowledge that the mechanism how BoNT could affect the central activity is not fully elucidated. Marchand-Pauvert et al. (2013) summarize the recent evidence of blockade of the gamma motor endings, of plastic changes following blockade of the neuromuscular transmission and of retrograde transport and transcytosis. Presently, it is not clear, which of these mechanisms contributes to the changes observed in functional imaging studies.

We acknowledge several limitations of the study which temper our conclusions and should be addressed in future research: recording the number of finger sequences completed per block does not capture all aspects of motor performance. The results should be replicated in a larger patient cohort, possibly using several different motor tasks, so that effects of a specific, carefully controlled and/or monitored motor task and a specific patient cohort might be separated. More timepoints post-treatment from baseline would permit better insight as to whether the changes in fMRI occurred before, after or at the same time as the improvements in clinical parameters—this may help explain whether the central changes are in fact primary driving the improvement or secondary effects. We acknowledge that the controls were scanned only once, whereas a balanced design would be more powerful to rule out effects of repeated motor testing. Nevertheless, single repetition of a motor task typically leads to a decrease, rather than increase, in sensorimotor activation (Hok et al. 2017 + další mimoOl citace). MRI-compatible electromyographic recording from the cervical musculature would permit modeling the influences of possible changes in dystonic activity after BoNT treatment. Finally, combining multiple examination methods in the same protocol, e.g., functional MRI and TMS, would provide richer data to help describe the complex pathophysiological processes (de Vries et al. 2012).

In conclusion, the results of the present study demonstrate that in treatment of CD, the first BoNT injection is associated with changes in widespread sensorimotor networks, which diminish the observed baseline differences between the patients and healthy controls. This study also confirms that abnormalities in sensorimotor activation extend beyond circuits controlling the affected body parts in CD.
